# Changes in the Patterns of Emergency Ambulance Care During a Primary Care Model Programme in Hungary

**DOI:** 10.3390/healthcare14081058

**Published:** 2026-04-16

**Authors:** Bernadett Szilágyi, János Sándor, Zoltán Ónodi-Szűcs, Karolina Kósa

**Affiliations:** 1 Faculty of Medicine, University of Debrecen, 4032 Debrecen, Hungary; sandor.janos@med.unideb.hu (J.S.); kosa.karolina@med.unideb.hu (K.K.); 2 Chancellery, University of Debrecen, 4032 Debrecen, Hungary; onodiszucs.zoltan@med.unideb.hu

**Keywords:** primary care, public health-focused primary health care, emergency ambulance care, global burden of disease, treatment outcome

## Abstract

Background: Hungary operated a public health-focused primary care model programme with expanded preventive and community-based services between 2013 and 2017 in four disadvantaged regions. This study aimed at assessing the association of this programme in one region with the patterns of emergency ambulance care before (2012) the programme and 3 years later when all services were available (2016). Methods: Patients in the selected region who received emergency ambulance care in the hospital catchment area were included. De-identified demographic data, reason for emergency service, on-site and hospital diagnosis, and treatment outcomes were entered into an electronic database from paper-based records. Diagnoses were assigned separate codes at GBD 1 and 3 levels. Results: The proportion of patients in emergency ambulance care showed a significant, 0.85% increase (*p* = 0.013) from 2012 to 2016. The proportion of female/male patients was roughly equal, but males needed emergency ambulance care significantly, 7 years younger than females in both years. Among patients with GPs in the model programme, 3.41% fewer needed emergency ambulance care due to non-communicable diseases, and 1.98% fewer were referred to other institutions from the hospital A&ED compared to those whose GPs did not participate (*p* < 0.001 for all). Conclusions: Utilisation of emergency ambulance services rose in the region in line with global trends suggesting that expanding primary care services alone may not be sufficient to reduce demand for emergency ambulance services. Further research is warranted to identify individual and systemic factors with major influence on emergency care use, including patient-level differences in the use of acute and preventive primary care services, and the availability of primary care after work hours.

## 1. Background

A persistent set of problems in emergency care results from inappropriate use (overuse) of emergency services that tends to be due to lack or difficulty of access to primary care, on one hand, and immediate availability of medical specialists, high technical level of diagnostics, and shorter waiting times on the other [1,2,3,4]. Lack of access to primary care is a major contributor to the overutilization of emergency care described among migrants compared to natives of countries in the European Economic Area [5,6]. But medically unjustifiable visits were found to be quite high even among citizens with access to primary care. In France, 14–27% [7], and in Romania up to 72% [8], of emergency visits were found to be inappropriate and could have been resolved in primary care.

Medically unjustified visits to emergency service providers contribute to longer waiting times, wasting resources, and lower quality of patient-centred care, and may even lead to overuse of health services, resulting in a wide range of long-term unfavourable consequences [9,10].

As much as emergency care is an essential domain of healthcare services, it cannot and should not supplant other forms of care, most importantly primary care. According to the World Health Organization [11], primary care workers as first-line responders should be able not only to manage health emergencies but also to provide some of the essential public health functions [12] such as protecting against health hazards, preventing or detecting diseases in their early stages, improving health service quality, and promoting health and well-being by engaging and educating local communities. Community engagement, empowerment and health education should optimally include communication about when to use emergency services [13].

This ideal of primary care with a broad range of disease-preventing and health-promoting services providing health for all was first defined in the Alma-Ata Declaration and reaffirmed by subsequent WHO documents [14]. However, its implementation has proven to be challenging, even for developed countries [15].

In alignment with WHO principles for prevention-oriented primary care, Hungary implemented a community-oriented model programme to widen the range of primary care services in 2013 in four disadvantaged regions of the country [16]. The five-year programme with substantial funding from the governments of Switzerland and Hungary created group practices called GP clusters. In addition to usual care, GP clusters, using the extra funding, launched previously unavailable services for local populations such as health status assessment, lifestyle counselling, and community health-promoting activities with the aim of increasing preventive service uptake and reducing inequality [17].

Since primary care in an optimal scenario can decrease the need for emergency care, our aim was to investigate changes in the volume of and patterns of those utilising ambulance emergency care in one geographical area of one GP cluster (Berettyóújfalu) out of the four GP clusters created in the model programme. We also supposed that hospital outcomes would change favourably when all preventive services were available. Data from two years were analysed to compare ambulance emergency care in 2012 (before the start of the programme) with data from 2016 when all elements of the programme, including all new services, were fully implemented and available in all GP clusters during the entire year.

## 2. Methods

### 2.1. Emergency Ambulance Care in Hungary

Emergency medical services (EMS) in Hungary are provided by the state to everyone, irrespective of citizenship and health insurance status, based on the Hungarian act on health (Healthcare Act of 1997). Patients can visit GPs at emergency clinics for emergency care, or they can go to centralised accident and emergency departments (A&ED) operating in hospitals [18]. Emergency care can also be requested by phone at the actual location of the patient and is typically provided by paramedics. Our analysis focused on this latter service modality, that is, emergency ambulance care provided by the National Ambulance Service (NAS) [19]. The NAS is divided into 7 regional units that operated 231 stations in 2013 [20]. Ambulance emergency services may provide definitive care on-site or transport the patient under care to the closest hospital A&ED for further care. Hospital care may entail 5 possible outcomes: (1) definitive care with subsequent discharge, (2) temporary observation for up to 24 h, (3) surgery, (4) admittance to inpatient care, or (5) referral to another healthcare institution.

The organisation and management of emergency ambulance care was no different in 2012 and 2016 as this type of care was not included in the model programme. Each step in the process of emergency ambulance care was documented on paper, along with patient personal details, the cause of call, on-site diagnosis and hospital diagnosis (in cases where the patient was transported there). All these documents were paper-based until 2017, when documentation was shifted to electronic devices [21]. Features of the procedure of emergency ambulance care are shown in Table 1.

### 2.2. Sources of Data

Data were obtained from the hospital based on previous agreements and ethical permission from all interested parties. A written agreement was signed by the researchers and the management of the hospital, according to which the paper-based records of ambulance emergency care could be studied in a selected room of the hospital, but no document was allowed to be copied, photographed or taken out of the hospital, and no information allowing for personal identification could be copied in any form.

The documents of patients who required emergency care and were transported by ambulance service to the Berettyóújfalu Hospital were available in the hospital. Paper-based documents were selected by hospital administrators for all emergency care patients in the years 2012 and 2016 whose residence was recorded in one of 36 settlements which belonged to the catchment area of the hospital. Each piece of demographic (age, sex, residence) and care-related data on the selected paper-based hospital records was entered manually into a database by the first author in the hospital under supervision.

Hospital documents for each patient included 2 documents: The first was an Ambulance Patient Care Report that contained demographic data, the location where the patient was found, cause of call, date and time of dispatch, time of arrival on scene, name and rank of the ambulance officer, response type of the ambulance, anamnesis, status, examination, applied treatment, summary, a statistical code used by the NAS, and on-site diagnosis without ICD code. This report was filled by the ambulance staff and handed over to the hospital upon arrival with the patient. The other document was the Emergency Inpatient Discharge Report with demographic information, the patient’s history, and additional information related to diagnostic and therapeutic procedures implemented in the hospital. This record also contained the diagnosis, with its ICD code, and further instructions. The total population numbers of the settlements within the catchment area for each year was obtained from the Central Statistical Office of Hungary.

### 2.3. Data Entry and Coding

The following data were entered manually into the electronic database. Cause of call, on-site diagnosis and hospital diagnosis were entered as stated on paper; then after checking and cleaning, each of the 3 definitions of health problems was allocated into one of the 3 broad cause groups of the Global Burden of Disease Project [22] (Level I: Communicable, maternal, neonatal and nutritional diseases; II: non-communicable diseases; III: injuries) and coded as separate variables. On-site diagnoses and hospital diagnoses were also allocated into an appropriate Level 3 subgroup specified by the Global Burden of Disease Project as separate variables.

The cause of call, on-site diagnosis and hospital diagnosis in the records were coded by the first author using the Diseases and Injuries Causes List of the World Health Organization’s Global Burden of Disease Protocol [23] (except when missing). Coding was checked by the last author. In cases of ambiguity, records were jointly discussed and re-evaluated using all available clinical information.

Based on the permanent residence of the patient, each record was coded as to whether it was included in the model programme (yes if the address belonged to GPs in the model programme; no if it was not). Also using the permanent residence, records were coded as to whether they were from a settlement with residentially segregated areas as defined by the Central Statistical Office (yes or no) [24]. Coding was performed by the first author and reviewed by the second author.

The type of hospital care was allocated into one of 5 categories: definitive outpatient care and discharge, observation, surgery, inpatient care (admittance to the hospital), and referral to another institution.

### 2.4. Statistical Analysis

Descriptive statistical analysis was carried out by using the t-test and analysis of variance for interval variables and the chi-square test for categorical variables. Poisson regression was performed to identify determinants—including gender, year, participation of GP in the model programme, and age when in emergency ambulance care—of the outcome variable, the number of cases in which emergency ambulance service was requested and patients were transported to hospital. Missing values are shown where relevant. Neither imputation nor sensitivity analysis was performed. MS Excel version number 365 and Stata BE version number 19 were used for statistical analysis, except for Poisson regression, which was performed by IBM SPSS Statistics version number 23 software. The alluvial and Sankey diagrams were prepared by SankeyEngine 1.0.1.0. (Visual Analytics Ltd., London, UK).

## 3. Results

Altogether 16 617 patients, or 18.32% of the total population in the covered area, in 2012 and 19 441 patients, or 22.10% of the total population, in 2016 received emergency care within the hospital’s catchment area, which represented a 3.78% increase from 2012 to 2016 (*p* < 0.001). Of those, 20.18% in 2012 and 21.03% in 2016 were transported by the National Ambulance Service, showing a significant increase (2012: 3.70%; 2016: 4.65% of the total population; *p* = 0.013). The rest of the patients arrived by other means of transportation. Of all patients transported by the NAS in the given year, only a small proportion (2.83% in 2012 and 2.52% in 2016) were below the age of 18 years, with no change between the 2 years (*p* = 0.403). The proportion of female children significantly increased from one-third to almost half of all patients under 18 years of age from 2012 to 2016. The percent of females significantly decreased by 2.63% from 2012 to 2016. The female/male ratio did not show significant change from 2012 to 2016. Males receiving emergency ambulance care were more than 7 years younger in both years compared to females (2012: d = 7.93 years; 2016: d = 7.24 years; *p* < 0.001 for both years) (Table 2). The number of patients in emergency ambulance care increased by 20.00% among those whose GP was in the model programme and by 22.21% among those whose GP was not (*p* = 0.792).

In terms of the mean age among those who received ambulance emergency care according to whether they had a GP in the model programme or not, there was no significant change in either group from 2012 to 2016.

Causes of calls were given by persons who requested emergency ambulance services, most frequently lay persons. The percent of missing causes of calls decreased significantly, by 2.33%, by 2016. More alarmingly, almost 12% of the on-site diagnoses (established by the staff of the ambulance) were missing in 2012, but this significantly decreased by 2016, and only a very small fraction of hospital diagnoses were missing in both years. In terms of diagnoses by GBD main groups, the largest, 3.63% increase was seen in the percent of calls due to non-communicable diseases, reflected in similar increases in the on-site (3.97%) and hospital diagnoses (3.13%). Less than a 0.8% increase was seen in communicable diseases, whereas emergency ambulance care required due to injuries decreased (Table 3).

**Figure 1:**
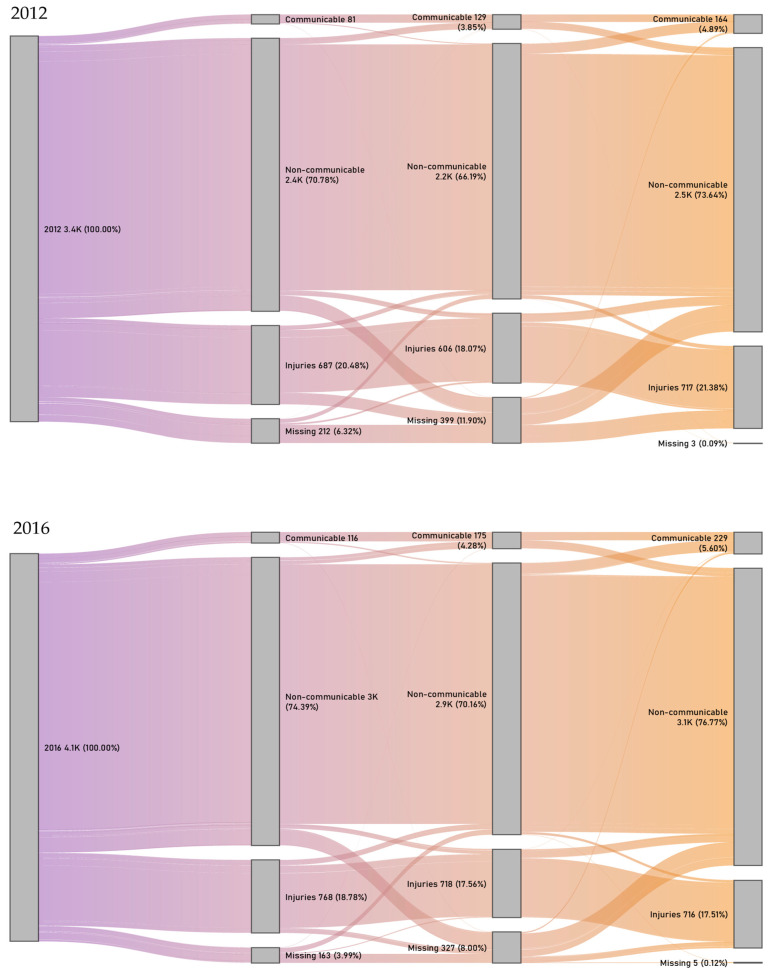
Alluvial diagrams showing all patients in emergency ambulance care (1st node) and their distribution by GBD broad cause groups according to cause of call (2nd node), on-site diagnosis (3rd node) and hospital diagnosis (4th node) in 2012 (**top**) and 2016 (**bottom**).

Of the four nodes of the alluvial diagrams for 2012 (Figure 1 2012 top panel) and 2016 (Figure 1 2016 bottom panel), the first node shows the total number of patients in emergency ambulance care in the given year, the second node shows their distribution by cause for requesting emergency care (cause of call), the third node shows distribution by on-site diagnosis (assigned by paramedics), and the fourth node reflects distribution by hospital diagnosis (assigned by specialists), all coded at GBD Level I. The comparison of the identity of diagnoses established by the ambulance crew at the patient’s location (third node) and in the hospital by specialists (fourth node) revealed that 80.11% of the patients had both on-site and hospital diagnoses in the same GBD main group in 2012; that increased to 83.83% in 2016 (*p* < 0.001) (Figure 1).

Next, we analysed the distribution of hospital diagnoses in 2016 by GBD main category in patients whose GPs participated in the model programme compared to those whose GPs did not. Among patients with GPs in the model programme, 3.41% fewer needed emergency ambulance care due to non-communicable disease compared to those whose GPs did not participate; 0.51% more patients needed emergency care because of communicable causes, while 2.83% more patients needed this type of care due to injuries. The distribution of hospital diagnoses by GBD broad group was significantly different between patients whose GPs were in the programme compared to those whose GPs were not (Table 4).

**Figure 2:**
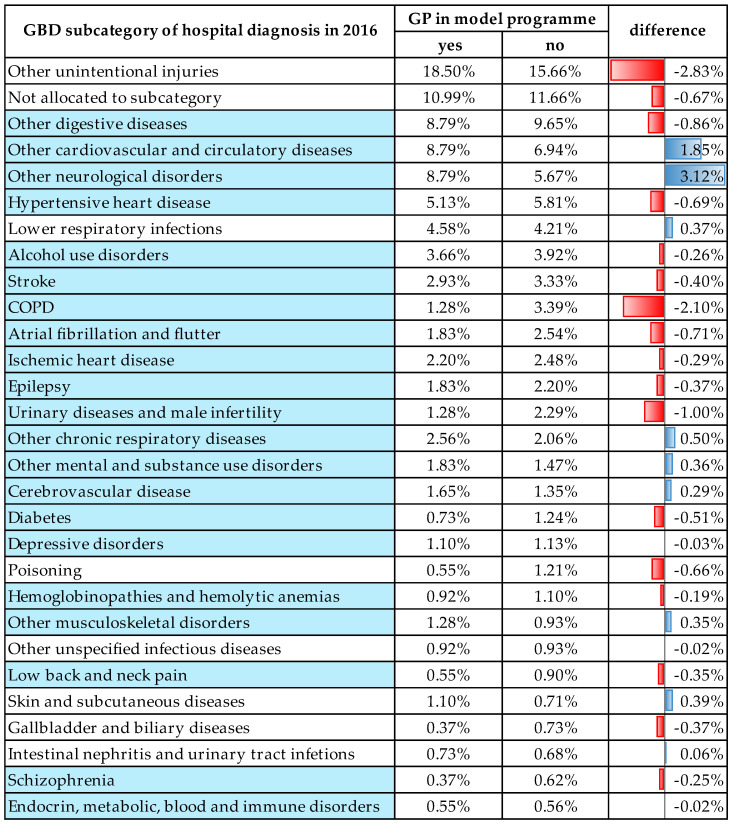
Distribution of hospital diagnosis by GBD subcategory of patients transported by the ambulance emergency service in 2016 with (yes) and without (no) their GP in the model programme.

The distribution of hospital diagnoses by subcategory of non-communicable causes between 2012 and 2016 was found to be significantly different (*p* < 0.001). Hospital diagnosis in 2016 was also compared between patients whose GPs participated in the model programme compared to those whose GPs did not. Figure 2 shows the proportion of hospital diagnoses in decreasing order in each GBD subcategory in which at least 20 cases occurred in the given year. Diagnoses in the non-communicable GBD subcategory are shown in blue. The difference in percent is colour-coded so that longer blue bars identify diagnostic subcategories which were more frequently identified in patients with GPs in the model programme, while red bars show GBD subcategories more frequently identified in patients of GPs not in the programme. The chi-square test was used to compare the distribution of the two groups of patients, which revealed a significant difference in distribution (*p* < 0.001)

**Figure 3:**
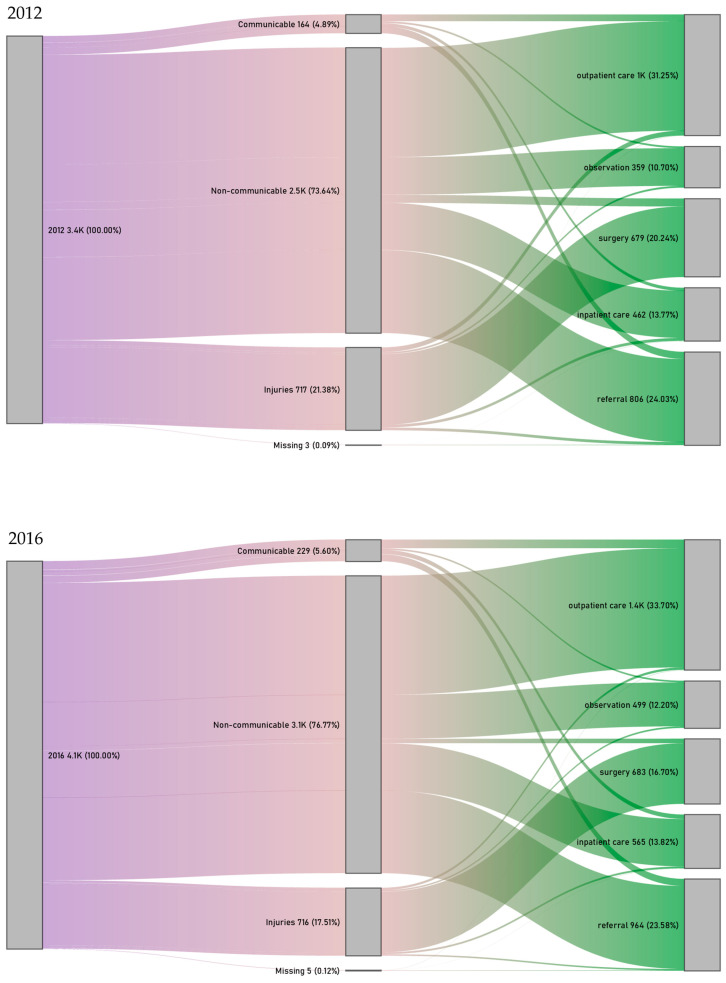
Sankey diagram of all patients in emergency ambulance care (1st node), the distribution of their hospital diagnosis by GBD main group (2nd node), and the type of hospital care they received (3rd node) in 2012 (**top**) and 2016 (**bottom**).

Lastly, we investigated the distribution of the final outcomes of ambulance emergency care that patients transported by the NAS received in the hospital. The first nodes of Figure 3 show the total number of patients transported by emergency ambulance into the hospital in the given year, the second nodes show their distribution by hospital diagnosis, and the third nodes show the type of outcome of hospital care. Of the five potential outcomes described in the Methods Section, the most frequent type of care in the hospital A&ED was outpatient care and subsequent discharge; approximately one-third of the patients received this type of care in both years (2012: 31.25%; 2016: 33.70%). The second most frequent type of care was referral to another institution (2012: 24.03%; 2016: 23.58%) followed by surgery (2012: 20.24%; 2016: 16.70%). Admittance to the hospital for inpatient care (2012: 13.77%; 2016: 13.82%), and observation for up to 24 h (2012: 10.70%; 2016: 12.20%) was provided to approximately one-eighth of the patients in both years (Figure 3).

We also analysed the type of care provided to ambulance emergency patients with non-communicable disease (based on the hospital diagnosis) in 2016 between those whose GPs participated in the model programme versus those whose GP did not. There were significant differences in the types of care these patients received, of which the most remarkable was that almost 2% fewer patients with GPs in the model programme were referred to another institution. Contrasting the types of care entailing a longer duration (inpatient care and referral) with short-term and/or definitive types of care (outpatient care with discharge, observation up to 24 h, surgery), significantly more (57.32%) patients with GPs in the model programme received short-term and/or definitive care, versus 56.21% of patients without GPs in the programme (*p* < 0.001) (Table 5).

## 4. Discussion

Our aim was to analyse potential changes in the patterns of ambulance emergency care between one year before (2012) and in another year (2016) after the implementation of a primary care model programme when all the programme’s extended services were available in a disadvantaged region of Hungary. The aims, organisation, services, and results of the model programme have been published elsewhere [16,17,24,25], but this is the first study that has addressed the question of whether the model programme, which offered extra prevention-focused services in addition to usual care, in one geographical area (out of four in the programme) had any relation to changes in the patterns of emergency ambulance care in the same region.

Results of the overall pattern of emergency ambulance care have shown that there was a 21% increase in the number of patients from 2012 to 2016 in the specified area; males needed emergency ambulance care approximately 7 years younger than females in both years; three-quarters of patients needed ambulance care because of reasons related to non-communicable diseases, and up to one-fifth because of injuries. A total of 80% of the on-site diagnoses (assigned by paramedics) and the hospital diagnoses (assigned by oxyologists in the hospital A&ED) were identical in terms of GBD main group, and the identity of these types of diagnoses significantly increased by almost 4% in 2016. The single most frequent GBD subcategory for ambulance emergency care was unintentional injury, followed by various cardiovascular causes. When comparing patients whose GPs participated in the model programme (so their patients had access to all extra services) to those who did not, no significant difference was found regarding patient numbers in emergency ambulance care between 2012 and 2016. However, we did find that significantly fewer patients with GPs in the model programme needed emergency ambulance care due to non-communicable diseases, though the most frequent causes in this group were still related to cardiovascular diseases, and significantly fewer patients with GPs in the model programme were referred to other institutions from the hospital A&ED. However, as these changes are small, more detailed analysis would be required to prove or disprove their practical relevance. Poisson regression taking into account the joint effect of the studied year and being in the model programme found no significant effect of the model programme on the risk of needing emergency ambulance care.

Due to the quasi-experimental design of the model programme, causal relationships between primary care services and outcomes cannot be established, only alluded to. Among other shortcomings, it must be mentioned that the analysis was restricted to one region (one GP cluster) out of a total of four clusters in the model programme since the investigated GP cluster operated in a region which had only one hospital A&ED in the area. Therefore, patterns seen in this region may be different from those in other regions of the country. An additional limitation may be caused by allocation bias due to the fact that patients with and without GPs in the model programme were identified by the street of their permanent residence. This might not have reflected their actual access to services in the model programme if their actual residence was different from that of their official permanent residence, or if they chose another GP using the legal possibility of selecting a GP different from the one allocated by permanent address. Since it is more likely that a patient would transfer to a GP who provided extra services than the other way around, this bias might have caused underestimation of the association between having a GP in the programme and emergency care patterns. However, these transfers tend to be very rare in general. Due to the quasi-experimental nature of the model programme and the fact that community health-promoting programmes within its framework involved a wide range of local residents (even those whose GPs were not in the programme), leakage or contamination (uncontrolled flow of intervention effects, e.g., health communication or patient education) might have occurred that would have reduced differences between patients in or out of the model programme.

However, the present study has several advantages, the first being that it was based on a comprehensive electronic database created from paper records for the investigated 2 years. To the best of our knowledge, there are no similar data in an electronic format in Hungary, since electronic recording in the National Ambulance Service was introduced in 2018 [21], and no similar analysis has been carried out for the model programme. The study was restricted to emergency ambulance care patients transported to hospital by the NAS so as to eliminate self-referrals from the sample. Another advantage of our study is that due to general coverage of health insurance in Hungary and the selected geographical location (mostly rural settlements in a disadvantaged region), there was a negligible number of persons who tend to use emergency care services at a disproportionately high rate (those without health insurance, the homeless, migrant persons). In addition, the investigated period (2012–2016) preceded the global outbreak of the COVID-19 pandemic; therefore, the results may serve as a useful pre-pandemic reference when interpreting more recent trends in emergency ambulance utilisation.

Similar studies on ambulance services in the country using data from after the introduction of electronic documentation have found that amenable mortality rates—that is, causes of death that could be avoided by appropriate medical care and ambulance services—showed considerable differences between counties in Hungary, particularly in acute myocardial infarction and haemorrhagic and ischemic stroke [26]. In our study, cardiovascular and other circulatory diseases constituted the third most frequent GBD subcategory for hospital emergency care, while stroke was the ninth most frequent cause. The former was more frequently diagnosed in patients with GPs in the model programme, and for the latter the opposite was true as shown in Figure 2. The variable quality of and unequal geographical access to emergency services seems to be a longstanding problem of the National Ambulance Service, the solving of which would require a comprehensive system of monitoring and evaluation [18,27,28].

The fundamental issue that our paper aims to address is the relation between primary care and emergency care, for which there is no clear-cut answer. One relevant WHO document emphasises the importance of strengthening primary care to improve emergency risk management, but it focuses on national (rather than individual) health emergencies [11].

Spanish authors found an increase in the number of emergency visits both in primary care centres and hospital accident and emergency departments between 1992 and 1997, and integrated analysis of time series revealed a long-term relationship between the two modalities of visits but did not provide evidence that emergency care at primary care centres would decrease visits to hospital A&E departments [29]. Increasing numbers of visits to emergency departments between 2003 and 2009, with the majority of patients not requiring hospital care, were also reported in the US [30].

On the other hand, British authors addressing the relation between the provision of primary care and emergency care found that referrals by general practitioners to one A&ED in London did show a decrease after reforms in primary care, but there was no decrease in self-referrals of nonlocal residents, homeless persons and others who had no access to primary care in London [31]. Others also found evidence that access to convenience-oriented primary care reduced the number of visits to hospital emergency departments [32]. Similarly, a positive impact on hospital A&ED visits was found in the UK when a pilot programme of 7-day opening of GP offices was introduced [33].

Based on these findings, the most reasonable conclusion seems to be that while prevention-oriented primary care is of fundamental importance, its effect on emergency care is strongly influenced by other factors, among them the availability of services beyond usual work hours. However, extending the availability of primary care services seems to be a real challenge given the worldwide shortage of primary care physicians [34,35,36] and healthcare workers [37] and EU-wide limitations on working time [38].

## 5. Conclusions

A public health-focused primary care model programme in a disadvantaged Hungarian region did not reduce overall emergency ambulance service utilisation over a 3-year period. Patients registered with GPs participating in the model programme required emergency ambulance care for non-communicable diseases and hospital referrals significantly less often than those outside the programme. Expanding primary care services alone may be insufficient to curb emergency care demand, highlighting the need for access to primary care after-hours and to address other systemic factors.

## Figures and Tables

**Table 1:** Features of emergency ambulance care in Hungary.

*Service Agency*	National Ambulance Service (NAS)		Hospital Emergency Department
*Type of service*	Dispatch based on call for emergency care	On-site care OR On-site care + transfer to hospital	Emergency medical care
*Record*	Dispatch Letter	Ambulance Patient Care Report	Emergency Inpatient Discharge Report
*Specification of medical problem*	Cause of call	Cause of call On-site diagnosis	Hospital diagnosis

**Table 2:** Demographic characteristics of emergency service patients transported by the NAS to the accident and emergency department of the hospital.

Reference: Patients in emergency care transported by the NAS to the hospital (N)	**2012**	**2016**	***p***
	3354	4089	
ALL PATIENTS			
Females % (N)	52.06 (1746)	49.96 (2043)	0.072
Mean age of ALL patients (year ± SD)	61.02 (±20.52)	61.51 (±20.27)	0.302
Mean age of ALL females (year ± SD)	64.82 (±19.18)	65.13 (±19.94)	0.627
Mean age of ALL males (year ± SD)	56.89 (±21.13)	57.89 (±19.95)	0.224
CHILDREN			
Persons under 18 years of age % (N)	2.83 (95)	2.52 (103)	0.403
Females under 18 years of age % (N)	33.68 (32)	48.54 (50)	**0.034**
ADULTS			
Persons over 17 years of age of ALL (%)	97.07	97.45	0.317
--Females over 17 years of age % (N)	52.64 (1714)	50.01 (1993)	**0.026**
--Males over 17 years of age % (N)	47.36 (1542)	49.99 (1992)	**0.026**
Mean age of adult patients (year ± SD)	62.48 (±18.89)	62.77 (±18.91)	0.516
Mean age of adult females (year ± SD)	65.82 (±17.86)	66.46 (±18.29)	0.283
Mean age of adult males (year ± SD)	58.77 (±19.32)	59.07 (±18.80)	0.642

Bold indicates statistically significant results (*p* < 0.05).

**Table 3:** Distribution of causes of call, on-site diagnosis and hospital diagnosis of patients requiring ambulance emergency service by GBD broad cause groups.

Types of Diagnoses	**2012**	**2016**	***p***
Cause of call			
I. Communicable, maternal, neonatal, nutritional diseases % (N)	2.42 (81)	2.84 (116)	<0.001
II. Non-communicable diseases % (N)	70.78 (2374)	74.39 (3042)	
III. Injuries % (N)	20.48 (687)	18.78 (768)	
Missing % (N)	6.32 (212)	3.99 (163)	<0.001
On-site diagnosis			
I. Communicable, maternal, neonatal and nutritional diseases % (N)	3.84 (129)	4.28 (175)	<0.001
II. Non-communicable diseases % (N)	66.19 (2220)	70.16 (2869)	
III. Injuries % (N)	18.06 (606)	17.56 (718)	
Missing % (N)	11.90 (399)	8.00 (327)	<0.001
Hospital diagnosis			
I. Communicable, maternal, neonatal and nutritional diseases % (N)	4.89 (164)	5.60 (229)	<0.001
II. Non-communicable diseases % (N)	73.65 (2470)	76.77 (3139)	
III. Injuries % (N)	21.37 (717)	17.51 (716)	
Missing % (N)	0.09 (3)	0.13 (5)	0.667

**Table 4:** Distribution of hospital diagnosis by GBD broad cause groups of patients transported by ambulance emergency service in 2016 with (yes) and without (no) their GP in the model programme.

GBD Broad Group	GP in Model Programme		*p*
	Yes	No	
I. Communicable, maternal, neonatal, nutritional diseases % (N)	6.04 (33)	5.53 (196)	<0.001
II. Non-communicable diseases % (N)	73.81 (403)	77.22 (2736)	
III. Injuries % (N)	19.96 (109)	17.13 (607)	

**Table 5:** Distribution of the outcomes (type of care received in the hospital) in patients with non-communicable hospital diagnosis according to patients’ status in the model project (yes or no) in 2016.

Hospital Diagnosis by GBD Main Group	Type of Care in Hospital A&ED % (N)	GP in Model Programme		Δ	*p*
		Yes	No		
Non-communicable disease	outpatient care	41.19 (166)	39.99 (1094)	1.21%	<0.001
	observation	14.14 (57)	14.88 (407)	−0.73%	
	surgery	1.99 (8)	1.35 (37)	0.63%	
	inpatient care	16.63 (67)	15.75 (431)	0.87%	
	referral	26.05 (105)	28.03 (767)	−1.98%	

## Data Availability

The datasets generated and/or analysed during the current study are available from the corresponding author on reasonable request, with the approval of the Director of Gróf Tisza István Hospital, Berettyóújfalu, Hungary. The dataset used in this study contains sensitive health-related information derived from patient records. Due to data protection regulations (including GDPR) and institutional policies, the data cannot be made publicly available.

## Abbreviations

The following abbreviations are used in this manuscript:

A&ED	Accident and Emergency Department
EMS	Emergency Medical Services
GBD	Global Burden of Disease
GP	General Practitioner
ICD	International Classification of Diseases
NAS	National Ambulance Service
